# Configuration of policy tools for promoting national fitness infrastructure supply: evidence from China

**DOI:** 10.3389/fpubh.2026.1822175

**Published:** 2026-06-10

**Authors:** Ziwen Zhang, Shun Wang, Xunling Wang

**Affiliations:** School of Physical Education, Huai Bei Normal University, Huaibei, China

**Keywords:** Fuzzy Set Qualitative Comparative Analysis, Latent Dirichlet Allocation, national fitness, Necessary Condition Analysis, policy tools

## Abstract

**Introduction:**

Against the backdrop of significant regional disparities in China’s public fitness infrastructure provision, this study aims to deeply analyze the diverse pathways and complex mechanisms driving high-level infrastructure supply from the perspective of policy tool combinations.

**Methods:**

This study utilizes a sample of 88 national fitness policy documents obtained from the 31 provincial-level administrative regions of China, covering the period from 2021 to 2025. It employs a mixed-methods approach: initially, the Latent Dirichlet Allocation (LDA) model is used to objectively identify policy instruments. Subsequently, a combination of Necessity Condition Analysis (NCA) and Fuzzy Set Qualitative Comparative Analysis (fsQCA) is applied to examine the bottleneck constraints and the configuration effects of various policy instruments on infrastructure supply.

**Results:**

The analysis of the LDA model identified five principal policy instruments: improve the public service system, organize scientific fitness guidance, build smart sports facilities, enhance fitness media outreach, and provide financial support. NCA indicated that no individual policy instrument is a necessary condition for the high-level supply of infrastructure, suggesting that isolated measures may be insufficient to overcome supply constraints. fsQCA uncovered three effective pathways that drive high-level supply, which can be categorized into two typical models: the “combined soft and hard measures” foundation-based path and the “public service-led” multi-element collaborative path. The former underscores the integration of scientific fitness guidance (soft) with the construction of intelligent facilities (hard), whereas the latter reflects comprehensive coordination of various resources centered on the public service system.

**Discussion:**

The provision of advanced national fitness infrastructure results from the coordinated implementation of multiple policy instruments. Governments at all levels should transition from reliance on isolated policies to adopting flexible deployment strategies tailored to their resource endowments, thereby improving the overall governance effectiveness of policy tools through the organic integration of technological empowerment, service optimization, and institutional safeguards.

## 1 Introduction

In recent years, global public health systems have encountered progressively severe structural challenges. Chronic noncommunicable diseases have become the primary source of the global disease burden (1), while inadequate physical activity is acknowledged as one of the principal behavioral risk factors influencing public health (2). Whether residents engage in physical activity is influenced not only by individual willingness but also significantly by the availability of sports infrastructure in their environment. Public fitness facilities such as sports venues (3), public fitness equipment (4), and sports parks (5) are widely recognized as key external factors influencing exercise participation rates. Accessible and diverse public sports facilities significantly enhance residents’ physical activity levels (6, 7), with particularly pronounced effects on middle-aged and older adults, women, and vulnerable groups (8–10).

### 1.1 Global public health context

With increasing urbanization and lifestyle modifications, sedentary behavior has become more widespread, thereby intensifying the global trend of insufficient physical activity. Data published by the World Health Organization (WHO) in 2024 indicates that approximately 31% of adults currently do not meet the recommended levels of physical activity, with projections suggesting this figure will rise to 35% by 2030. The continual rise in global physical inactivity underscores the importance of promoting universal engagement in physical exercise, which has become a shared objective of public health governance on an international scale (11). To alleviate public health pressures and elevate national physical activity levels, national strategies such as the U.S. Physical Activity Guidelines, the UK’s Everybody Active, Every Day, the Japan’s National Sports Promotion Plan and the China’s fitness policy all emphasize fostering more active lifestyles through enhanced sports facility provision and improved public health literacy.

### 1.2 Policy instrument theory and research gaps

Policy instruments are generally characterized as the amalgamation of diverse means and techniques utilized by governmental authorities to achieve policy aims, with the intention of influencing social behavior and policy results. The traditional definition by Vendung et al (44). perceives policy instruments as a collection of techniques used for exercising governmental authority to promote or hinder social change (12). In early research, German economist Kirschen (13) pioneered the systematic categorization of policy instruments, proposing a list encompassing sixty-four policy means. While his work laid the groundwork for subsequent analyses, it lacked a clear classification framework or operational guidance (13). Subsequently, Hughes simplified Kirschen’s framework, categorizing policy instruments into four fundamental types: supply, subsidy, production, and regulation (14). As policy research advanced, scholars increasingly classified policy instruments based on the degree of government intervention. Howlett et al. (15) distinguished among voluntary, coercive, and hybrid types according to the level of compulsion inherent in policy implementation, emphasizing the diversity of governmental engagement approaches. Schneider and Ingram, focusing on policy impact mechanisms, observed that policy instruments possess not only institutional attributes but also value orientations, manifesting in forms such as incentive-based, persuasive, and capacity-building approaches (16). Rothwell and Zegveld’s (45) “supply–demand-environment” tripartite framework gained widespread adoption due to its clear structure and strong applicability, becoming a mainstream analytical framework, particularly in Chinese sports policy instrument research (17). This classification highlights the supportive role of policy instruments at various stages towards policy objectives, facilitating systematic mapping of government intervention pathways. Furthermore, McDonald and Elmore categorize policy instruments based on implementation goals into directive, incentive-based, capacity-building, and systemic change types, underscoring their mediating function between policy objectives and implementation outcomes (18). So far, policy instrument theory studies present two notable limitations in the identification of policy tools: firstly, a heavy dependence on subjective manual coding of policy texts (19), employing traditional classifications such as supply-oriented, environment-oriented, demand-oriented (11, 17), or command-oriented, incentive-oriented, and information-oriented (20, 21); secondly, their analytical perspectives are predominantly static, often emphasizing the isolated effects of individual policy tools while neglecting the combined characteristics and the dynamic evolution of various tools during actual implementation.

### 1.3 China’s national fitness policy

Before the 2008 Beijing Olympics, China’s longstanding emphasis on elite sports exerted pressure on mass sports initiatives. In the post 2008 period, the concept of “sports for all” gradually gained prominence, leading to incremental adjustments in national fitness policies in terms of organizational structures, regulations, and budgets. This transformative trajectory is closely linked to policy decisions influenced by political elites (22).

National fitness policies have significantly contributed to increasing physical activity levels and preventing non-communicable diseases by improving the built environment, enhancing public sports facilities, and promoting active transportation. These effects are especially evident in the development of community fitness spaces and green parks (23). However, policy outcomes exhibit significant heterogeneity across regions and demographic groups, reflecting variations in sports human capital and public fiscal healthcare expenditure, and highlighting a strong dependence on regional developmental foundations and the structure of public resource allocation (24).

Some research has formulated a national fitness development index system, providing quantitative tools for assessing policy performance (25). Community-level studies indicate that policy implementation has markedly improved residents’ exercise conditions; however, facility utilization efficiency and scientific guidance remain inadequate (26). Studies based on national sports venue censuses and community field investigations reveal that persistent urban–rural disparities, insufficient public accessibility, and low spatial utilization efficiency remain key challenges hindering public participation in sports and limiting policy effectiveness (27, 28). Studies during the pandemic emphasize the critical importance of national fitness policies in strengthening national health resilience and overall governance capabilities (29).

Scholars have systematically analyzed policy documents related to national fitness from 1995 to 2025. Existing research largely focused on facility supply and demand (30, 31), construction models (32, 33), and factors influencing resident behavior (34, 35). The structure of policy instruments exhibits certain imbalances, with environmental and supply-side tools prevailing, while demand-side tools remain relatively insufficient. Although overall policy consistency is high, opportunities for further optimization exist (11). To our knowledge, existing studies lack systematic approaches to uncover the combined and dynamic interactions among policy tools.

### 1.4 Objective

This study aims to address two key issues in policy instrument theory by developing an objective classification of public health policy instruments and investigating the “multi-path synergistic mechanisms” through which different combinations of policy tools enhance public fitness supply capacity. To this end, the study addresses two research questions: (1) How can public health policy instruments be objectively classified using a data-driven approach? (2) How do different policy tool combinations for national fitness influence infrastructure supply levels?

## 2 Materials and methods

### 2.1 Study design

**Figure 1:**
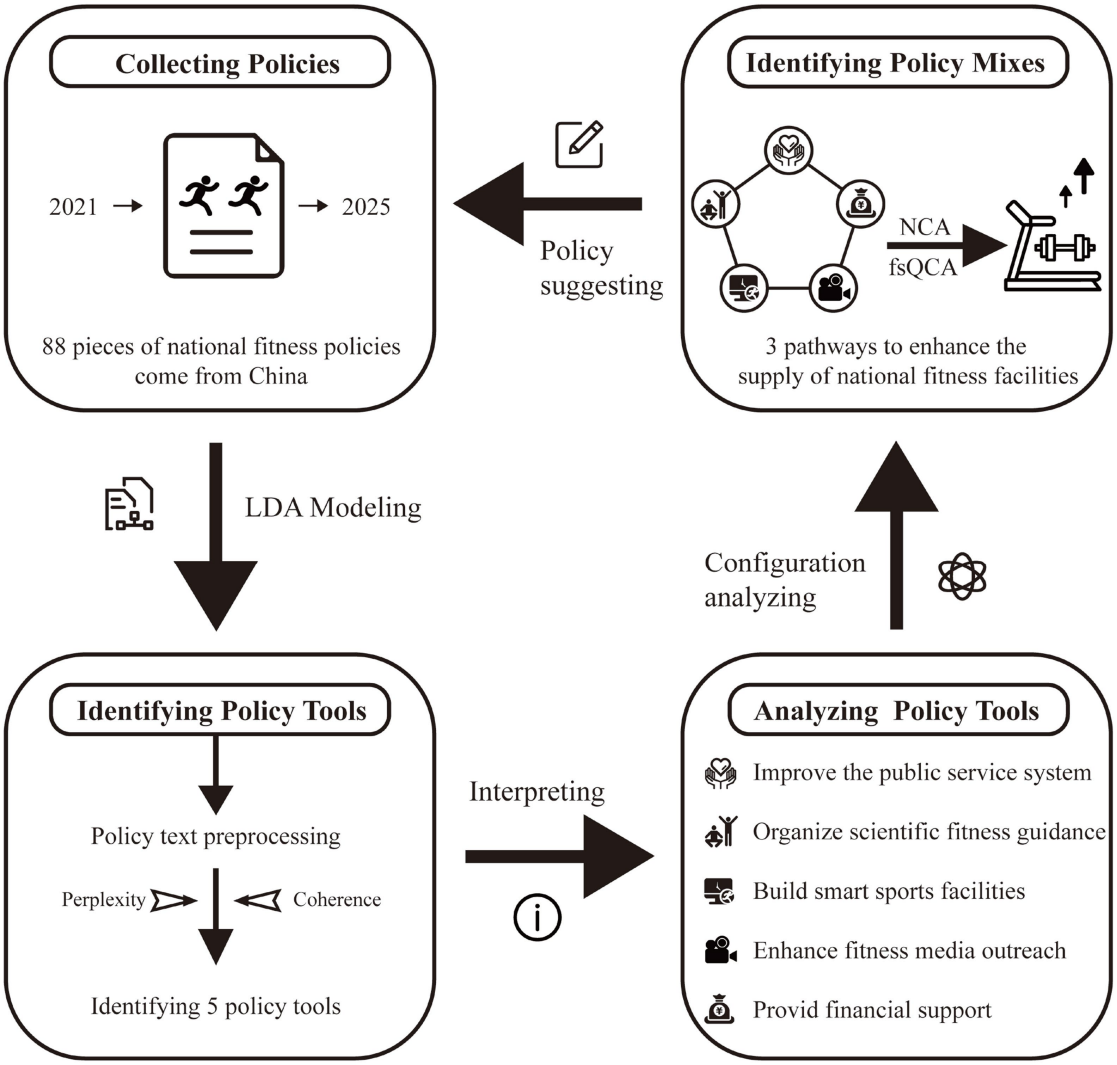
Research design diagram illustrating the mixed-methods analytical framework.

This study adopts an integrated methodology that combines quantitative policy text identification with configurational causal analysis. The specific design is illustrated in Figure 1.

### 2.2 Settings

China’s National Fitness Program is a long-term public health policy framework (1995–2030) to promote widespread physical activity and population health, implemented through cycles of successive five-year plans since 2011. The study focuses on 2021–2025 cycle.

### 2.3 Document selection

Provincial-level policy documents were collected through cross-referencing and supplementary searches within the 2021–2025 cycle. The search was conducted using the keywords “fitness” and “national fitness” across three sources. First, the Peking University Law Database, a comprehensive and authoritative legal information repository in China, was utilized to retrieve officially promulgated provincial sports and public health regulations. Second, the official websites of provincial governments and sports administrative departments provided direct access to the most recent government notices, implementation plans, and departmental directives. Finally, the Baidu search engine served as a supplementary tool to cross-verify the collected documents and ensure no relevant local policy announcements were omitted. The collection of national fitness policy texts adheres to the “triangular cross-verification” principle.

### 2.4 Preprocessing

During data preprocessing, the Jieba Tokenizer in Python was employed for Chinese text segmentation. To ensure reproducibility and meaningful topic differentiation, a rigorous data cleaning pipeline was implemented. First, standard stop words were eliminated utilizing the widely adopted Harbin Institute of Technology (HIT) stop word list. Second, given the domain-specific nature of our policy corpus, we supplemented the standard list with a custom dictionary of high-frequency administrative and policy-related terms (e.g., “government,” “national,” “policy,” “province,” and “notice”). As these ubiquitous terms possess low discriminative power in this specific context, their explicit removal was crucial to prevent them from obscuring the latent thematic structures. Finally, standard filtering steps were applied: single-character tokens were removed due to their lack of independent semantic meaning, and extreme document frequency thresholds were set. Specifically, terms appearing in fewer than 3 documents (min_df) or in more than 90% of the documents (max_df) were filtered out to eliminate rare words and corpus-specific stop words. This comprehensive preprocessing strategy yielded a refined, high-quality vocabulary suitable for robust LDA modeling.

### 2.5 Variables

The outcome is the level of national fitness infrastructure provision (NFIP). The condition variables are macro-categories of the policy instruments derived from the document corpus.

### 2.6 Data source and measurement

NFIP is measured by the absolute aggregate count of physical fitness amenities within each province. Data were extracted from the “Fitness Map” section of the National Fitness Information Service Platform,^1^ maintained by the General Administration of Sport of China. Specifically, this metric is calculated as the unweighted sum of three primary categories of actively operating infrastructure: public sports venues, pieces of public fitness equipment, and sports parks. We selected this absolute aggregate metric because it intuitively reflects the macro-level gross volume and tangible physical output of sports public service provision in each region, serving as a reliable foundational benchmark for fsQCA calibration.

Macro-categories of policy instruments are operationalized as the topic scores derived through LDA applied to the selected corpus of 88 documents (as detailed in Section 2.3).

### 2.7 Analytical framework

The analytical procedure consists of three sequential stages. First, the LDA model was utilized to extract topics from the policy texts, with the optimal number of topics determined using perplexity and coherence metrics. Second, the NCA was employed to identify whether specific policy tools constitute prerequisites for achieving high-level national fitness infrastructure provision. Finally, fsQCA was conducted to examine how different combinations of policy tools contribute to the outcome. NCA and fsQCA offer complementarity analytical perspectives and theoretical emphases: the former highlights constraints relationships, whereas the latter captures configurational patterns of causal conditions.

#### 2.7.1 Latent Dirichlet Allocation

LDA is an unsupervised topic modeling technique founded on a probabilistic Bayesian generative mechanism, extensively utilized in the analysis of policy texts and government documents (36). Its core principle assumes that documents are composed of multiple underlying themes, each weighted differently, whereby each theme consists of a set of words with high topic-word probabilities demonstrating stable co-occurrence patterns. The model starts by specifying the number *K* of topics and setting Dirichlet priors over document–topic (with hyperparameter *α*) and topic–word (with hyperparameter *β*) distributions. The hyperparameter *β* was set to 0.1, while for *α*, we utilized the alpha = ‘auto’ option in the gensim package to learn an asymmetric prior from the data. To ensure the stability of the extracted topics, we conducted sensitivity analyses by testing multiple random seeds (42, 100, 200, 500, and 1,000) and extending the search range of candidate values up to *K* = 15. Through an iterative estimation procedure, the model repeatedly updates the topic assignment probabilities of words based on how prevalent each topic is in the document and how strongly each word is associated with the topic. These updates rely on count-based probabilities derived from the data. Over many iterations (100 in our case), the assignments stabilize, revealing coherent topic structures. For each province, the topic score was computed as the sum of the document-topic probabilities *p*(*z_k_*|*d*) across all documents belonging to that province. LDA can autonomously identify latent thematic structures within large-scale policy texts without dependence on researcher-imposed classifications, thereby effectively mitigating subjective biases inherent in manual coding (37). The optimal number of topics was selected by evaluating perplexity and coherence scores across a range of candidate values (*K* = 1, …, 15), choosing the value that minimized perplexity while maximizing coherence.

Finally, by analyzing the top-10 keywords ranked by their topic-word probability, we identified and refined the types of policy instruments represented by each topic. Python was employed to conduct LDA analysis.

#### 2.7.2 Necessary Condition Analysis

The NCA identifies the constraining effects of condition variables on outcome variables by plotting ceiling lines. This primarily involves two techniques: Ceiling Regression (CR) and Ceiling Envelopment (CE) under a freely disposable shell. The CR method is more suitable when the independent variable is continuous or a discrete variable with five or more levels; while the CE method is better suited for binary variables or discrete variables with fewer levels. Given the actual measurement characteristics of the study variables, to enhance the robustness of results and the credibility of interpretations, this paper reports the analysis results of both CR and CE methods simultaneously. When the two methods yield differing conclusions regarding necessity, the CR method’s conclusion is prioritized because both the condition variables (thematic intensities of policy tools) and the outcome variable NFIP are continuous numerical variables, for which the CR method provides more robust and reliable estimates. To rigorously quantify this necessity constraint, NCA calculates the effect size (*d*), which represents the proportion of the ceiling zone (*C*) relative to the entire empirical scope (*S*). This relationship is defined mathematically as shown in Equation 1:


$$d=\frac{C}{S}$$ (1)

where the *C* is the area above the ceiling line indicating the region of necessity constraint, and the *S* is the total possible area of observations. A variable is deemed a necessary condition only when the effect size (*d*) exceeds 0.1, the result is statistically significant (*p* < 0.01), and accuracy exceeds 95% (38). Effect sizes are interpreted as follows: 0.1 ≤ *d* < 0.3 (small effect), 0.3 ≤ *d* < 0.5 (medium effect), and *d* ≥ 0.5 (large effect).

R software was employed to conduct NCA single-factor necessity analysis, examining whether the policy instruments identified through the LDA constitute necessary conditions for enhancing the supply level of national fitness infrastructure.

Based on the analysis of necessary conditions, and given the unique advantage of NCA in identifying “bottleneck levels,” a further bottleneck level analysis was conducted to characterize the minimum levels that each explanatory condition must satisfy at different levels of achievement of the outcome variable.

#### 2.7.3 Fuzzy Set Qualitative Comparative Analysis

Unlike conventional linear regression analysis, which disregards configurational effects, fsQCA utilizes set-theoretic logic to integrate qualitative case insights with quantitative analysis, emphasizing “multiple concurrent causation” and “path dependence” phenomena. The primary strength of this methodology resides in its capacity to identify “equifinality”—the notion that different pathways can lead to the same outcome—by demonstrating how various condition configurations yield identical results. Through the analysis of set membership rather than simple correlations, it reveals asymmetric causal relationships between variables (39), thereby accurately identifying diverse effective pathways that contribute to high-quality public service provision.

To objectively evaluate the validity of these pathways, fsQCA relies on two core set-theoretic parameters: consistency and coverage. Consistency measures the degree to which instances of *X* are a subset of *Y*, representing the reliability of the causal pathway. Coverage evaluates the empirical relevance of the configuration by measuring the proportion of *Y* that is explained by *X*. Consistency and coverage are calculated using Equations 2 and 3, respectively:


$$\text{Consistency}\;(X\leq Y)=\frac{\sum \min(X_{i},Y_{i})}{\sum X_{i}}$$ (2)

$$\begin{array}{c} \text{Coverage}\;(X\leq Y)=\frac{\sum \min(X_{i},Y_{i})}{\sum Y_{i}} \end{array}$$ (3)

where *X_i_* represents the calibrated membership score of case *i* in the condition configuration, and *Y_i_* represents its calibrated membership score in the outcome.

In addition to configurational sufficiency, fsQCA can also be employed to test necessary conditions, although its analytical logic and criteria differ substantially from NCA. NCA adopts a “constraint-bottleneck” perspective, concentrating on identifying indispensable preconditions for outcome generation. It emphasizes the minimum threshold constraints established by conditional variables across various outcome levels, quantifying necessity through effect sizes and bottleneck levels. Conversely, fsQCA’s necessity testing is founded on set-theoretic logic, assessing whether a particular set of conditions consistently forms a superset of the outcome set—that is, whether cases within the outcome set necessarily belong to the condition set in a set-theoretic sense. Integrating both approaches facilitates the cross-validation of necessary conditions across various levels of causal logic, thereby bolstering the reliability of research conclusions. For this purpose, fsQCA evaluates the universal applicability of a single condition across all cases with high or low outcomes, generally establishing a consistency threshold above 0.9 as the rigorous criterion for defining a necessary condition (40).

The fsQCA 4.1 software was utilized to systematically investigate multiple configuration pathways that achieve high levels of public fitness infrastructure provision. In the course of analysis, considering the sample size characteristics and established research practices, the case frequency threshold was set at 1, and the consistency threshold at 0.8. Furthermore, acknowledging the substantial variation in the Proportional Reduction in Inconsistency (PRI) across different experimental contexts, as well as its distribution exhibiting distinct breakpoint characteristics, the PRI threshold was established at 0.8 via the natural breakpoint method. This value surpasses the lower standard of 0.6 commonly employed in prior studies (41), thereby effectively reducing potential conflicting configurations and improving the robustness of the results interpretation.

Concerning the identification of configuration results, the core and peripheral conditions across various configurations were distinguished through a comparative analysis of intermediate solutions and simplified solutions. Conditions that appeared in both intermediate and simplified solutions were characterized as core conditions, signifying their crucial role in the formation of configurations. Conversely, conditions present solely in intermediate solutions and absent from simplified solutions were designated as peripheral conditions, highlighting their auxiliary influence on outcomes when specific circumstances are met.

### 2.8 Data calibration

The fundamental principle of fsQCA involves calibrating raw variables into set membership scores ranging from 0 to 1, thereby delineating each case’s degree of association with specific condition or outcome sets. The critical aspect of this process is the proper establishment of calibration anchors, which facilitate the interpretation of variable values as belonging to either “high-level” or “low-level” sets. Building upon established research paradigms (42), the “direct calibration” methodology is employed. Considering the actual distribution characteristics of the sample data, the 95th, 50th, and 5th percentiles of variable values in the sample are designated as calibration standards for “Full membership,” “Crossover point,” and “Full non-membership,” respectively. This approach helps to mitigate potential distortions caused by extreme values during calibration, thereby enhancing comparability across different cases. Furthermore, given that membership values of 0.5 may influence the stability of set identification in logical operations, we adhered to established research practices by adding a constant of 0.001 to all calibrations. The specific calibration anchors and their corresponding values, along with the descriptive statistics for each variable, are presented in Table 1.

**Table 1:** Calibration and descriptive statistics of the outcome and variables derived from LDA applied on Chinese national fitness policy documents (2021–2025).

Variable type	Variable name	Calibration anchors			Mean	SD	Min	Max
		Full membership	Crossover point	Full non-membership				
Outcome variable	Level of public fitness infrastructure provision (Count of facilities)	60525.000	14396.000	2266.600	20201.290	21427.306	535.000	101599.000
Conditional variables (LDA intensity scores)	Improve the public service system	2.730	0.470	0.001	0.876	0.966	0.000	3.341
	Organize scientific fitness guidance	1.635	0.030	0.001	0.373	0.829	0.000	4.165
	Build smart sports facilities	1.521	0.030	0.001	0.301	0.532	0.000	1.988
	Enhance fitness media outreach	1.176	0.044	0.001	0.250	0.496	0.000	2.152
	Provide financial support	2.144	0.147	0.001	0.645	1.049	0.000	5.019

### 2.9 Robustness assessment strategy

Following established QCA research practices, the robustness of the configurational results was assessed by adjusting the consistency threshold. Specifically, the consistency threshold was increased from 0.80 to 0.85 to examine whether the identified configurations remained stable under a stricter criterion (43). In addition, the stability of the LDA topic model was assessed through sensitivity analyses using multiple random seeds and an extended candidate topic range from *K* = 1 to *K* = 15.

## 3 Results

### 3.1 Corpus characteristics

The corpus consists of 88 national fitness policy documents collected from 31 Chinese provinces. After rigorous preprocessing—including text segmentation, stop-word removal, and term filtering—the corpus retains a total of 185,420 tokens, with a unique vocabulary size of 8,652 words. The document lengths vary, with an average length of 2,107 tokens, ranging from a minimum of 645 to a maximum of 5,120 tokens.

### 3.2 Policy tool extraction based on LDA topic model

**Figure 2:**
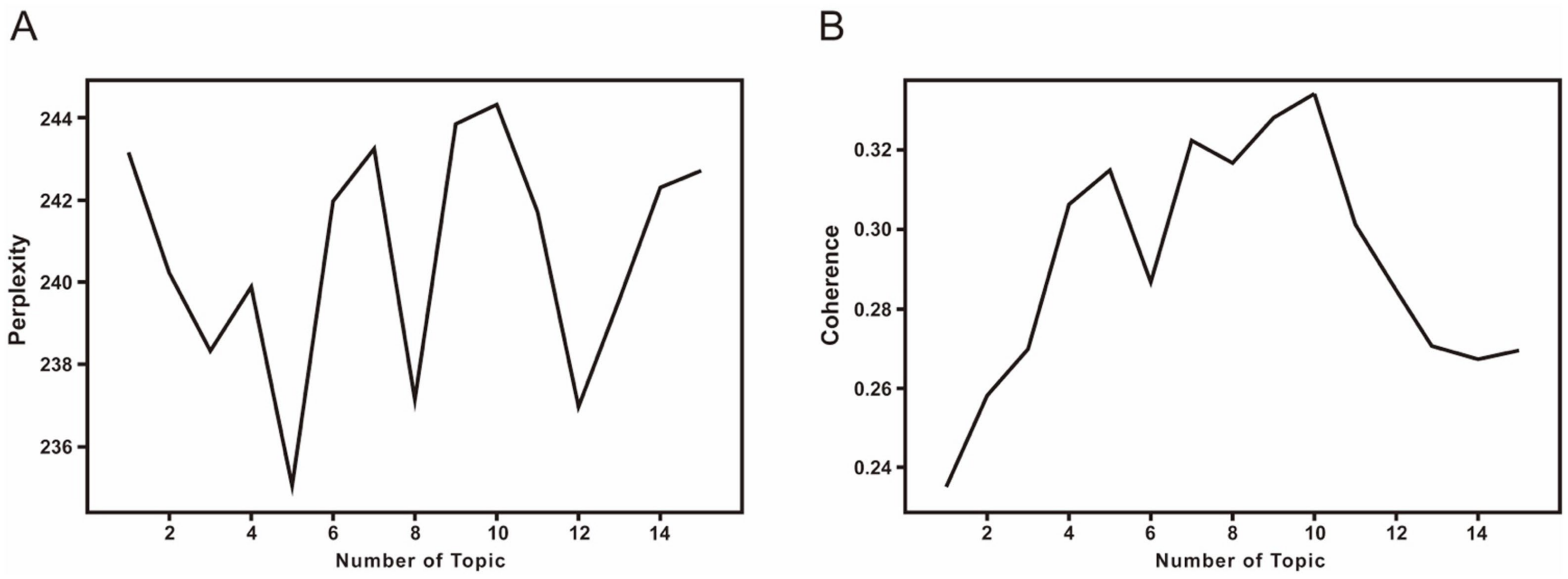
Perplexity **(A)** and coherence **(B)** scores of the LDA model across candidate topic numbers from *K* = 1 to *K* = 15, used to determine the optimal number of policy tool topics from the national fitness policy corpus.

As illustrated in Figure 2, the perplexity value reaches its global minimum at five topics, indicating the best model fit at this level. The coherence score remains relatively high and stable at five topics, indicating strong semantic consistency among the keywords within each topic. Based on these metrics, the optimal number of topics is identified as five, corresponding to five distinct policy instrument categories. The asymmetric document-topic prior parameters estimated using gensim’s alpha = ‘auto’ option were 0.15, 0.22, 0.18, 0.25, and 0.20 for Topics 1–5, respectively. These values indicate that Topic 4 and Topic 2 were relatively more prevalent across the corpus, whereas Topic 1 was more concentrated in a smaller subset of documents.

Based on the top 10 words with the highest topic-specific probabilities, the five topics were interpreted as five policy instrument categories. Table 2 presents the most representative terms and their probability weights for Topics 1 to 5, which provide the empirical basis for naming and interpreting the policy tools.

**Table 2:** Top 10 words with high topic-specific probabilities (weights) and corresponding policy tool categorizations derived from the LDA of 88 provincial national fitness policy documents.

Topic 1		Topic 2		Topic 3		Topic 4		Topic 5	
Topic 10 words	Weights	Topic 10 words	Weights	Topic 10 words	Weights	Topic 10 words	Weights	Topic 10 words	Weights
Public	0.011	Collaborative	0.006	Data	0.022	Enhance	0.007	Introduction	0.008
Science	0.009	Organization	0.006	Digital	0.018	Online	0.007	Grant	0.008
Supply	0.009	Teaching	0.006	Digitalization	0.013	Propaganda	0.006	Public	0.007
Services	0.008	Scientific	0.005	Systems	0.012	Promotion	0.006	Investment	0.007
Standardization	0.007	Layout	0.005	Intelligence	0.010	Protection	0.005	System	0.006
Collaborative	0.007	Guidance	0.005	Big Data	0.010	Media	0.005	Ten Thousand Yuan	0.006
Governance	0.006	Instruction	0.005	Infrastructure	0.009	Fitness	0.005	Funding	0.006
Modernization	0.005	Positioning	0.005	Construction	0.007	Health	0.005	Fiscal	0.005
Improvement	0.005	Masses	0.005	Introduction	0.007	Exercise	0.005	Capital	0.005
Coverage	0.005	All Citizens	0.005	Intelligent	0.007	Communication	0.005	Economy	0.005

Policy tool 1: improve the public service system (IPSS). This policy tool focuses on enhancing the public service system for national fitness, emphasizing the government’s responsibility for coordinated planning and its role in ensuring basic service provision. Key terms like “Public,” “Supply,” and “Services” indicate that this tool aims to broaden the coverage and spatial accessibility of national fitness infrastructure by expanding public service provision and optimizing service structure and quality. “Science” and “Standardization” reflect the emphasis on scientific planning and institutional norms during policy implementation. Keywords like “Collaborative” and ‘Governance’ indicate a focus on multi-stakeholder coordination and optimizing governance mechanisms. “Modernization,” “Improvement,” and “Coverage” further demonstrate that this tool is oriented toward modernizing the public service system, providing institutional support to elevate the supply level of national fitness infrastructure.

Policy tool 2: organize scientific fitness guidance (OSFG). This tool focuses on organized, professional fitness guidance to indirectly enhance infrastructure efficiency by improving service delivery capabilities. Words with high topic-specific probabilities terms like “Scientific,” “Guidance,” “Instruction,” and “Teaching” emphasize guiding residents to safely and effectively use fitness facilities through scientific exercise principles and standardized methods. Terms like “Organization,” “Collaborative,” and ‘Layout’ reflect the policy’s emphasis on systematically integrating fitness guidance resources and optimizing spatial layouts during implementation, facilitating the coordinated allocation of fitness guidance services and infrastructure development. “Masses,” “All Citizens,” and “Positioning” demonstrate this tool’s goal of universal coverage. By expanding the service radius of fitness guidance, it enhances the utilization efficiency and service performance of existing fitness infrastructure, thereby strengthening the practical effectiveness of infrastructure supply.

Policy tool 3: build smart sports facilities (BSSF). This tool focuses on elevating the quality tier and operational efficiency of national fitness infrastructure through digital and intelligent means. Words with high topic-specific probabilities terms like “Data,” “Digital,” “Digitalization,” and “Big Data” indicate the policy’s emphasis on embedding data elements into infrastructure construction and operation processes, enhancing the precision and dynamic responsiveness of facility provision through information collection and analysis; Terms like “Systems,” “Intelligence,” and ‘Intelligent’ reflect the policy’s reliance on smart systems to drive the transformation of fitness facilities from traditional supply models to intelligent operations. Concurrently, “Infrastructure,” “Construction,” and “Introduction” indicate that this tool optimizes the functional structure and service formats of public fitness infrastructure by introducing new technologies and models, thereby advancing infrastructure supply toward higher quality and efficiency.

Policy tool 4: enhance fitness media outreach (EFMO). This policy tool improves the alignment between public fitness infrastructure supply and resident demand by strengthening information dissemination and public opinion guidance mechanisms. “Enhance,” “Media,” “Online,” and “Communication” indicate that the policy leverages diversified communication platforms to elevate public awareness regarding fitness facility distribution, usage methods, and service offerings; “Propaganda” and “Promotion” reflect the policy’s cultivation of public fitness awareness and participation willingness through sustained advocacy, thereby unlocking latent demand for fitness infrastructure; “Fitness,” “Health,” and ‘Exercise’ demonstrate how this tool integrates health promotion objectives with infrastructure utilization; while “Protection” emphasizes safeguarding public access to information and accessibility for fitness activities. Overall, this policy tool facilitates effective alignment between fitness infrastructure supply and public usage through demand-side guidance, indirectly enhancing the allocation efficiency and social benefits of infrastructure provision.

Policy tool 5: provide financial support (PFS). This tool centers on fiscal guarantees and capital investment to deliver direct resource backing for national fitness infrastructure construction and operation. Words with high topic-specific probabilities terms like “Fiscal,” “Funding,” “Public,” and ‘Investment’ indicate that the policy increases investment in fitness venues, facilities, and supporting projects through government fiscal expenditures, special funds, and public investment. “Grant,” “Introduction,” and “Capital” reflect how the policy promotes social capital participation in infrastructure construction and broadens funding sources through subsidy mechanisms, guidance, and capital introduction. “System” and ‘Economy’ reflect the institutionalized management of capital allocation and its synergistic effects with regional economic development; while “Ten Thousand Yuan” indicates the policy’s emphasis on quantitative management and performance-oriented funding allocation at the implementation level. Overall, this policy tool directly enhances the construction capacity and supply scale of public fitness infrastructure by establishing a stable, diversified funding guarantee mechanism.

### 3.3 Single-factor necessity analysis

#### 3.3.1 NCA single-factor necessity analysis

Table 3 presents the results of the NCA single-factor necessity analysis. The effect sizes of IPSS, OSFG, BSSF, and EFMO are all below 0.1, indicating that none of these policy tools constitutes a necessary condition for achieving a high level of infrastructure supply. Although PFS shows an effect size above 0.1 and accuracy exceeding 95% under both CR and CE methods, its *p*-value is greater than 0.01, and therefore it does not meet the criteria for a necessary condition. Furthermore, the results of the bottleneck level analysis are presented in Table 4.

**Table 3:** NCA results evaluating the necessity of five individual policy instruments for achieving high level NFIP.

Conditions	Method	Accuracy	Ceiling zone	Scope	Effect size	*p*-value
IPSS	CR	93.5%	104398.833	337616.118	0.031	0.026
	CE	100%	132250.118	337616.118	0.040	0.022
OSFG	CR	93.5%	16096.641	420925.363	0.038	0.172
	CE	100%	15067.639	420925.363	0.036	0.208
BSSF	CR	96.8%	47994.289	200878.911	0.024	0.054
	CE	100%	48703.271	200878.911	0.023	0.059
EFMO	CR	93.5%	2842.453	217473.318	0.013	0.389
	CE	100%	4341.139	217473.318	0.020	0.341
PFS	CR	96.8%	157000.019	507233.910	0.310	0.017
	CE	100%	193438.670	507233.910	0.381	0.021

IPSS, improve the public service system; OSFG, organize scientific fitness guidance; BSSF, build smart sports facilities; EFMO, enhance fitness media outreach; PFS, provide financial support. CR, Ceiling Regression; CE, Ceiling Envelopment.

**Table 4:** Bottleneck level analysis results from the NCA, indicating the minimum required implementation levels (%) of each policy tool at varying targets of infrastructure supply.

NFIP	IPSS	OSFG	BSSF	EFMO	PFS
0	NN	NN	NN	NN	NN
10	NN	NN	NN	NN	NN
20	NN	NN	NN	0.2	NN
30	4.3	NN	NN	0.5	NN
40	15.7	NN	4.7	0.9	9.6
50	27	1.8	16.4	1.3	23.4
60	38.4	4.1	28	1.6	37.2
70	49.7	6.4	39.7	2	51
80	61.1	8.6	51.3	2.4	64.9
90	72.5	10.9	62.9	2.7	78.7
100	83.8	13.1	74.6	3.1	92.5

IPSS, improve the public service system; OSFG, organize scientific fitness guidance; BSSF, build smart sports facilities; EFMO, enhance fitness media outreach; PFS, provide financial support. NN indicates that the explanatory condition does not constitute a necessary constraint for a given outcome level.

#### 3.3.2 fsQCA single-factor necessity analysis

The fsQCA necessity test results (Table 5) show that the consistency values for all five policy tools remain below the 0.9 threshold under both high and low NFIP conditions. This indicates that no individual policy tool functions as a necessary condition for determining infrastructure supply levels. This finding is consistent with the NCA results, further supporting the robustness of the conclusion.

**Table 5:** fsQCA necessity test results, displaying the consistency and coverage metrics for single conditions under both high and low NFIP.

Sets of conditions	High NFIP		Low NFIP	
	Consistency	Coverage	Consistency	Coverage
IPSS	0.638	0.626	0.491	0.630
~IPSS	0.624	0.410	0.708	0.719
OSFG	0.692	0.727	0.485	0.665
~OSFG	0.681	0.503	0.801	0.773
BSSF	0.676	0.714	0.465	0.642
~BSSF	0.661	0.486	0.793	0.762
EFMO	0.644	0.671	0.510	0.695
~EFMO	0.707	0.525	0.758	0.735
PFS	0.749	0.525	0.445	0.559
~PFS	0.542	0.721	0.778	0.802

IPSS, improve the public service system; OSFG, organize scientific fitness guidance; BSSF, build smart sports facilities; EFMO, enhance fitness media outreach; PFS, provide financial support. ~ denotes the negation of the condition (low membership in the fuzzy set).

### 3.4 Configuration analysis

The fsQCA results (Table 6) identify three configurations that lead to a high level of infrastructure supply. The overall solution consistency is 0.918, indicating a high degree of reliability, and the solution coverage is 0.541, suggesting substantial explanatory power. The three configurations differ in their core and peripheral policy tool combinations, as discussed below.

**Table 6:** fsQCA results of sufficient configurations, revealing three distinct policy tool pathways (S_1_, S_2_, S_3_) driving high NFIP.

Antecedent variables	S_1_	S_2_	S_3_
IPSS		U	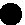
OSFG	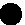	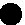	U
BSSF	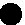	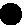	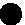
EFMO	U	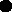	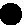
PFS	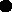		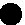
Consistency	0.966	0.952	0.915
Raw coverage	0.376	0.308	0.329
Unique coverage	0.062	0.023	0.093
Solution consistency	0.918		
Solution coverage	0.541		

IPSS, improve the public service system; OSFG, organize scientific fitness guidance; BSSF, build smart sports facilities; EFMO, enhance fitness media outreach; PFS, provide financial support. 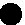 indicates the presence of a core condition; 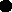 indicates the presence of a marginal condition; U indicates the absence of a core condition; “blank” means that this condition can either exist or not exist.

#### 3.4.1 “Combined soft and hard measures” foundation-based path

This pathway corresponds to configuration paths S_1_ and S_2_, which exhibit consistency coefficients of 0.966 and 0.952 respectively, thereby demonstrating strong explanatory power in enhancing the provision of high-level public fitness infrastructure. Although the two configuration pathways differ in peripheral conditions such as fiscal support and fitness media dissemination, they maintain high consistency in their core condition structures, both emphasizing OSFG and BSSF policy instruments. OSFG focuses on improving the safety and standardization of facility usage through institutionalized organization and professional guidance, while BSSF offers essential material infrastructure and technological support to facilitate precise scientific fitness guidance by incorporating digital and intelligent technologies and upgrading infrastructure functionality. These two instruments work synergistically, collectively establishing the “functional foundation” for the supply of national fitness infrastructure. This illustrates the fundamental principle of advancing infrastructure provision through coordinated “soft service delivery” and “hard facility construction.”

Building on this foundation, different cases can achieve path equivalence through differentiated auxiliary policy tools. Configuration S1 relies on PFS to continuously strengthen investments in smart sports facilities and professional guidance systems, thereby forming a resource-driven foundational supply model. Configuration S_2_ relies on EFMO to enhance public awareness and willingness to use fitness facilities and services, thereby stimulating demand-side factors and improving the utilization efficiency of infrastructure supply. Despite their different supporting conditions, both configurations share the same core logic of synergistically advancing scientific fitness guidance and smart sports facilities.

Using Anhui Province as a representative case within this pathway: Concerning the ‘Organizing Scientific Fitness Guidance,” Anhui has persistently advanced the development of a four-tier scientific fitness guidance service network encompassing provincial, municipal, county, and community levels, thereby establishing a relatively comprehensive vertically integrated system. By leveraging policy documents such as the Anhui Provincial Fitness Implementation Plan (2021–2025), the 2023 Implementation Plan for the Anhui Happy Fitness Initiative, and the Anhui Provincial Sports Bureau Notice on Promoting the Construction of Scientific Fitness Guidance Centers, the province has systematically cultivated its social sports instructor workforce and extended scientific fitness guidance services to grassroots levels. By the end of 2025, the province aims to have over 200,000 registered social sports instructors, achieving near-universal coverage across communities and administrative villages. Utilizing central funding, 103 public sports venues throughout the province provide residents with free or low-cost access, regularly conducting physical fitness assessments, prescribing exercise regimens, and instructing fitness skills. This integration of scientific fitness guidance into the daily operations of public fitness infrastructure significantly enhances facility safety and standardization.

Regarding the development of “building smart sports facilities,” the Five-Year Action Plan for Strengthening Anhui’s National Fitness Infrastructure (2021–2025) explicitly articulates that, by the conclusion of 2025, a minimum of 587 new intelligent fitness facilities—including advanced exercise equipment—will be installed, and in excess of 60 public sports venues will undergo comprehensive digitalization and informatization upgrades. Through the integration of smart fitness equipment, sports data collection systems, and online management platforms, real-time monitoring and meticulous management of facility operations and utilization behaviors are achieved. This initiative not only enhances the functional architecture of fitness venues but also ensures robust technical support for the accurate dissemination and dynamic adjustment of scientific fitness guidance.

Concerning funding guarantees, the Notice on Allocating 2023 Sports Power Province (Happy Fitness) Construction Funds explicitly assigns 150 million yuan in provincial special funds. Simultaneously, through provincial coordination, central budgetary funds and sports lottery public welfare funds are prioritized for grassroots sports facility construction and mass fitness events. At the local level, the role of sports lottery public welfare funds is fully harnessed, being directly allocated to the construction of fitness facilities, venue upgrades, and the provision of public sports services, thereby further strengthening the fiscal foundation for the development of national fitness infrastructure. This diversified and institutionalized funding support mechanism has effectively promoted the overall enhancement of Anhui Province’s capacity to supply national fitness infrastructure.

Through the implementation of a policy toolkit focused on “scientific fitness guidance + smart sports facilities,” Anhui Province has methodically augmented its capacity to supply public fitness infrastructure. This case illustrates that concurrently reinforcing “soft service capabilities” alongside “hard facility foundations,” supported by consistent fiscal guarantees, can create a highly replicable approach for the provision of foundational public fitness infrastructure.

#### 3.4.2 “Public service-led” multi-element collaborative path

This pathway corresponds to configuration path S_3_, with a consistency score of 0.915. It is centered on the policy tools IPSS, BSSF, EFMO, and PFS as core conditions. It constitutes an implementation pathway driven by the development of the public service system and the synergistic application of various policy tools. Among these, the IPSS policy tool, which exists as a core condition, indicates that institutional-level public service supply capacity serves as the foundational and supportive condition for achieving target outcomes. It provides institutional and organizational guarantees for the effective operation of other policy tools. The BSSF and EFMO policy instruments reinforce this pathway by enhancing both hardware facility provision and information dissemination mechanisms. By improving facility accessibility and awareness of fitness, they expand the coverage and practical impact of policy tools. The core presence of the PFS policy instrument further provides stable resource guarantees for refining the public service system, facility construction, and communication system operations, thereby strengthening the sustainability of the policy combination. Notably, while the OSFG policy tool exhibits a core condition deficiency within this pathway, this indicates that in scenarios characterized by an advanced public service system, high levels of facility intelligence, and adequate dissemination and funding support, specialized individual guidance is not an essential prerequisite for achieving outcomes. Its function does not serve as a critical enabling condition for results within this configuration.

Using the Inner Mongolia Autonomous Region as a representative example within this framework: the region has effectively enhanced the supply of national fitness infrastructure by improving public service systems, bolstering smart sports facilities and media dissemination mechanisms, and securing stable fiscal investment. According to the “Inner Mongolia Autonomous Region National Fitness Implementation Plan (2021–2025),” by 2025, public sports facilities at the county (city, district), township (subdistrict), and village (community) levels, as well as the “15-min fitness circle” in urban communities, will achieve comprehensive coverage. The per capita sports venue area is expected to increase to 2.6 square meters. The plan explicitly advocates for the development of a higher-level public service system for national fitness, addressing deficiencies through projects aimed at expanding venue facilities, enhancing service functions, and increasing digital service delivery capabilities. Institutional frameworks will support the sustainable development of the sports public service system.

Concerning the provision of institutional public services, as exemplified by Hohhot’s development of key sports parks and community fitness centers, the municipality has augmented and modernized numerous sports venues in recent years. The total number of sports facilities now amounts to 6,457, achieving comprehensive coverage of fitness amenities within communities and administrative villages. The per capita sports venue area has reached 2.72 square meters, and a fundamental public sports service network encompassing both urban and rural regions has been established. Furthermore, the city has proactively improved the accessibility and service capacity of sports facilities. By establishing a “10-min sports circle,” it has markedly diminished the spatial distance between residents and infrastructure, thereby significantly enhancing the coverage and convenience of public fitness facilities.

Regarding hardware facilities and information dissemination, the autonomous region has actively promoted free or low-cost access to public sports venues. Over 130 sports facilities in Inner Mongolia have been included in the list seeking central government funding support for free or low-cost public access, aiming to increase infrastructure supply and service utilization rates. Furthermore, the “Inner Mongolia Autonomous Region National Fitness Implementation Plan” proposes advancing the digitalization of public fitness services, establishing a mechanism for timely information dissemination and convenient service access, and creating a national fitness big data platform. This platform will support facility usage management, venue reservations, and event announcements, enhancing the convenience of resident participation and the efficiency of service provision.

At the fiscal level, the autonomous region has integrated its sports development plan for the 14th Five-Year Plan period with strengthened financial support measures. This provides sustained funding guarantees for public sports service delivery and the development of smart sports industries, driving the deep integration of the public sports service system with the region’s overall socioeconomic development.

Under the “public service-led” multi-factor coordination approach, Inner Mongolia has established an implementation mechanism based on the development of an institutional public service system. This mechanism is supported by the provision of intelligent sports facilities and media promotion, and is further reinforced by stable fiscal support. This combination of diverse policy instruments not only enhances the level of supply and accessibility of national fitness infrastructure but also fortifies the coordination between institutional frameworks and technological innovations, as well as the connection between supply and demand within the public sports service system. Such an approach significantly advances the high-quality development of the region’s sports sector.

### 3.5 Robustness and sensitivity analyses

The robustness and sensitivity analyses were conducted for both the LDA topic model and the fsQCA configurational results. For the LDA model, we re-estimated the topic model under multiple random seeds (42, 100, 200, 500, and 1,000) and evaluated candidate topic numbers from *K* = 1 to *K* = 15. Across these specifications, the five-topic structure remained substantively stable. The optimal solution was consistently located around *K* = 5, and the top keywords of the five topics showed high overlap across repeated estimations. For the fsQCA results, when the consistency threshold was increased from 0.80 to 0.85, the configurational solutions remained unchanged. The core condition combinations and the overall configuration structure showed no substantial variation under this stricter criterion, and the parameters of fit continued to meet established empirical standards. These findings confirm the robustness and stability of the empirical results.

## 4 Conclusion and implications

### 4.1 Research conclusions

This study systematically examined the complex relationship between public fitness policy instruments and infrastructure provision levels across China’s 31 provincial-level administrative regions. It integrated LDA thematic modeling, NCA, and fsQCA. Key findings are as follows:

First, utilizing the LDA thematic model, five central public fitness policy instruments were objectively discerned from a corpus of 88 policy documents covering the 2021–2025 period. These include: Improving the public service system, Organizing scientific fitness guidance, Building smart sports facilities, Enhancing fitness media outreach, and Providing financial support. This discovery indicates that governmental entities at various levels predominantly depend on five policy dimensions to promote national fitness development: institutional enhancement, technological empowerment, service provision, publicity mobilization, and financial safeguards.

Second, the NCA results demonstrate that none of the five policy tools is a necessary condition for the provision of high-level national fitness infrastructure. This suggests that isolated policy tools alone are insufficient to establish the prerequisites for advanced infrastructure supply, highlighting the importance of utilizing a combination of policy tools.

Third, the fsQCA configuration analysis identified three effective pathways driving high-level public fitness infrastructure provision, which were further categorized into two typical models: the “combined soft and hard measures” foundation-based path (including configurations S_1_ and S_2_) centers on “organize scientific fitness guidance” and “build smart sports facilities” as core conditions, emphasizing the deep integration of technological empowerment and professional services; The “public service-led” multi-element collaborative path (including configuration S_3_) builds upon “improve public service systems,” supplemented by building smart sports facilities, enhancing fitness media outreach, and providing financial support, demonstrating the holistic coordination of institutional strengths and diverse approaches. These pathways indicate that regions can achieve equivalent high-level supply through differentiated combinations of resources based on resource endowments and institutional environments.

The primary contributions of this study are reflected in the following four aspects: Methodologically, it integrates LDA, NCA, and QCA methods, introducing an objective and replicable pathway for identifying policy tools; theoretically, it applies policy instrument theory to the fields of national fitness and public sports service system development, expanding the explanatory power of relevant theories in sports governance. Empirically, it reveals the conditional configurations through which different policy instrument combinations enhance national fitness infrastructure supply from a provincial comparative perspective, providing new evidence for understanding the heterogeneous effects of local policies. Practically, it offers data-driven, structured decision-making support for governments to optimize policy design and combinations, thereby promoting the high-quality development of the national fitness public service system.

### 4.2 Managerial implications

Initially, governments should prioritize the comprehensive integration of “soft services” and “hard facilities,” considering smart technologies as a fundamental advancement for increasing supply efficiency. Research demonstrates that dependence solely on facility expansion cannot sustainably fulfill high-quality development requirements; therefore, “building smart sports facilities” must be closely coupled with “organizing scientific fitness guidance.” It is recommended that local governments focus not only on expanding physical infrastructure but also on concurrently implementing digital management systems and employing professional guidance personnel. This dual approach of “technology and service” will augment the utilization efficiency and service quality of existing facilities, thereby enhancing the overall effectiveness of infrastructure provision.

Secondly, fiscal funding support and media dissemination mechanisms serve as vital catalysts for the development of a high-level public service system. For regions that lack digital infrastructure or professional guidance resources, the institutional safety net of “improving public service systems” should be fully utilized, supplemented by consistent fiscal investment and widespread media outreach. This highlights that government policy formulation should prioritize creating a supportive social environment and ensuring adequate funding. By enhancing top-level design and promotional strategies, it can encourage social entities to participate in infrastructure development, thereby establishing a multi-stakeholder governance model for supply.

Finally, it is essential to recognize that no single policy tool can independently drive a leap in the supply level of national fitness infrastructure. NCA analysis did not identify any single necessary condition, while fsQCA further revealed pathways involving combinations of multiple policy tools. This indicates that high-level supply is more likely to result from the coordinated deployment of policy tools rather than isolated breakthroughs. Therefore, governments at all levels should abandon a “one-size-fits-all” policy mindset and instead implement systematic combination strategies. In practice, regions should flexibly select either the “combined soft and hard measures” foundation-based path or the “public service-led” multi-element collaborative path based on local economic foundations, technical conditions, and resident needs. Strengthening integration and collaboration across departments regarding funding, technology, talent, and promotional resources will achieve holistic governance and optimal effectiveness of policy tools.

### 4.3 Limitations and prospects

This study employs a mixed-methods design integrating LDA, NCA, and fsQCA to systematically elucidate the multifaceted driving mechanisms and configuration pathways through which policy tool combinations enhance the provision of public fitness infrastructure. It offers novel analytical perspectives for the optimization of sports policies. However, certain limitations persist.

Firstly, methodologically, while hyperparameter optimization (using the alpha = ‘auto’ option) and random seed sensitivity analyses were rigorously conducted for the LDA model, the coherence trajectory may still exhibit localized fluctuations due to the relatively concentrated size of the provincial policy corpus. Consequently, the 5-topic solution was ultimately retained for two critical reasons: it ensures optimal theoretical interpretability, and it strictly adheres to the mathematical constraints of fsQCA. Given our macro-level sample size of 31 provinces, selecting 5 policy instruments (condition variables) perfectly avoids the problem of “limited diversity” and excessive logical remainders, thereby ensuring the validity of the configurational pathways. However, because the sample is confined to these 31 provincial-level regions, the external validity of the findings is somewhat restricted. Future research could expand the corpus to include municipal, county, or community-level documents. This would not only yield even more statistically stable LDA topic estimates independent of macro-configurational constraints but also allow for cross-national comparisons to augment the universality of the conclusions.

Secondly, reliance on cross-sectional data from the 2021–2025 policy cycle poses challenges in fully capturing the dynamic evolution of policy tool mechanisms. Considering potential time lags between policy inputs and infrastructure provision, subsequent research could incorporate longitudinal data using panel fsQCA or time-series methods to more comprehensively reveal the long-term effects of policy tool combinations and their dynamic adjustment mechanisms.

Moreover, due to the inherent limitations of the fsQCA method, its capacity for causal interpretation of variables over time remains expandable. Future research could build upon the existing analytical framework by integrating emerging factors such as digital technology transformation and social capital participation. Exploring their moderating effects on the effectiveness of policy tool allocation would provide more forward-looking theoretical and empirical support for advancing the high-quality implementation of the national fitness strategy.

## Data Availability

The raw data supporting the conclusions of this article will be made available by the authors, without undue reservation.

## Footnotes

1
https://www.js365.org.cn/
